# Net platelet clot strength of thromboelastography platelet mapping assay for the identification of high on-treatment platelet reactivity in post-PCI patients

**DOI:** 10.1042/BSR20201346

**Published:** 2020-07-15

**Authors:** Daye Cheng, Shuo Zhao, Yiwen Hao

**Affiliations:** Transfusion Department, First Affiliated Hospital of China Medical University, Shenyang, China

**Keywords:** antiplatelet, high on treatment platelet reactivity, platelet mapping, thromboelastography

## Abstract

High-on treatment platelet reactivity (HTPR) leads to more prevalence of thrombotic event in patients undergoing percutaneous coronary interventions (PCI). Dual antiplatelet therapy with aspirin in addition to one P2Y_12_ inhibitor is commonly administrated to reduce HTPR. However, ‘one size fits all’ antiplatelet strategy is widely implemented due to lacking benefits with tailored strategy. One reason for the failure of tailored treatment might be less specificity of the current indicators for HTPR. Therefore, searching for specific indicators for HTPR is critical. Thromboelastograph with platelet mapping (TEGpm) assay has been explored for identifying HTRP. Variables of TEGpm assay, including maximum amplitude (MA) induced by thrombin (MAthrombin), R time, platelet aggregation rate induced by ADP (TEGaradp) and MA induced by ADP (MAadp) have been demonstrated to be able to identify HTPR in post-PCI patients. However, these variables for HTPR might be less specific. Thus, in the present study, a novel variable nMAadp was derived by removing fibrin contribution from MAadp and analyzed for its usefulness in determining HTPR. In addition, MAthrombin, *R* time, MAadp and TEGaradp were also examined for determining HTPR. In conclusion, nMAadp and TEGaradp were demonstrated to be independent indicators for HTPR; nMAadp had the strongest power to identify HTPR with cutoff value of 26.3 mm; MAthrombin and *R* time were not significantly different between patients with and without HTPR; combination of TEGaradp and nMAadp further improved the ability to identify HTPR with an AUC of 0.893.

## Introduction

Platelet activation and reactivity play pivotal roles in thrombosis [1–3]. Patients with acute coronary syndromes (ACS) and patients undergoing percutaneous coronary intervention (PCI) with higher platelet reactivity on-treatment (HTPR) are at more risk of occurrence of thrombotic events [4–6]. Thus, antiplatelet therapy with aspirin in addition to one P2Y_12_ pathway inhibitor (dual antiplatelet therapy, DAPT) has been widely introduced to decrease on-treatment platelet reactivity (TPR) aiming to reduce the risk of thrombotic event occurrence in patients after PCI [7]. However, ‘one size fits all’ antiplatelet strategy is currently administrated without discrimination of individually variable responsiveness to specific antiplatelet drugs and TPR [4,5], which might lead to the reduced efficacy of antiplatelet treatment. Therefore, personalized antiplatelet strategy based on assessment of individual responsiveness to antiplatelet medication and TPR should improve the clinical outcome theoretically, despite the multiple studies failing to show benefits from tailored treatment [4,8–10].

Platelet function tests (PFTs) are *ex vivo* tests used for the assessment of *in vivo* platelet function and TPR and have been extensively explored to identify the patients at high risk of adverse event occurrence [11]. Currently, a number of PFTs have been introduced, which can be classified into point-of-care test and laboratory based test. Laboratory-based assays mainly include light transmittance aggregometry (LTA) and vasodilator-stimulated phosphoprotein (VASP) phosphorylation; point-of-care tests (POCTs) consist of platelet function analyzer (PFA), VerifyNow and thromboelastography (TEG) modified with platelet mapping (TEGpm) [12,13]. An ideal PFT should be rapid, accurate, reliable and easy to operate as well as available conveniently [13]. LTA was developed in early 1960s, and has still been considered as a classical gold method to evaluate platelet reactivity [14]. However, LTA is characterized with lacking standardization, time-consuming, plasma based test, complicated sample preparation and professional expertise requirement, which limits its clinical routine application [15–17]. While the point-of-care test, TEGpm, meets the optimal PFT demands for clinical practice due to easy to perform, being available at bedside with flexible time and whole blood sample for testing.

Standard TEG was developed half a century ago and designed to measure the overall coagulation process with whole blood sample [18]. So far, TEG has been adopted broadly for monitoring hemostasis and guiding blood transfusion in surgical and traumatic patients [19–21]. TEG assesses coagulation process with measuring several parameters, including R time indicating thrombin initiation duration, maximum amplitude (MA) indicating maximum clot strength formed by crosslink of platelet and fibrin, and others, such as Ly30 indicating thrombolytic process [21,22]. However, standard TEG is not able to assess the specific effects of antiplatelet drugs directly. Thus, a modified TEG with platelet mapping assays, TEGpm, was introduced and explored to evaluate platelet reactivity and effect of antiplatelet drugs [23]. Essentially, TEGpm is able to measure four MA levels, including MA induced by thrombin stimulation (MAthrombin), MA induced by arachidonic acid (AA) (MAaa) or MA induced by adenosine diphosphate (ADP) (MAadp) and MA due to fibrin cross linking (MAfibrin), simultaneously and separately [24]. The presumption of TEGpm design is that equal levels of MAthrombin, MAaa and MAadp would be produced in the absence of any effect of antiplatelet drugs [24]. Conversely, any kind of reduction of MA level would be presumably attributed to the effect of specific antiplatelet drug, e.g. the reduction of MAaa due to COX-1 pathway inhibited by aspirin or the reduction of MAadp due to P2Y_12_ pathway inhibitors. To avoid the nonspecific interference of fibrin and reflect the specific contribution of platelet to the clot strength, platelet aggregation rate (TEGar) induced by specific agonists, was calculated by the following equation:

$$\text{TEGar}=\frac{\text{(MAadp}\;\text{or}\;\text{MAaa}\;\text{-}\;\text{MAfibrin)}}{\text{(MAthrombin}\;\text{-}\;\text{MAfibrin)}}\times 100$$

**Figure 1:**
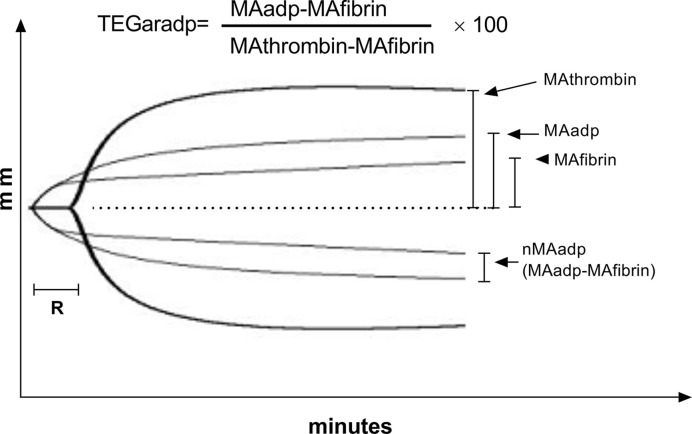
Curves show tracing of TEGpm with agonist of ADP Equation above the trace indicates the calculating expression of TEGaradp; TEGpm, thromboelastography with platelet mapping; TEGaradp, percentage of platelet aggregation rate induced by adenosine diphosphate (ADP) detected by TEGpm; MAthrombin, maximum amplitude (MA) induced by thrombin; MAadp, MA induced by ADP; MAfibrin, MA due to fibrin crosslink; *R*, time of coagulating factor reaction indicating thrombin generation; nMAadp, net MAadp.

[25], the depictive illustration of taking TEGar induced by ADP (TEGaradp) for example is shown in Figure 1.

Risk indicators to predict HTPR and adverse event occurrence are of great importance in clinical practice. Several parameters of TEGmp assay have been explored for evaluating HTPR and demonstrated to be potential risk indicators for adverse event occurrence in post-PCI patients by multiple studies [25–28]. In an early study, increased TEGar determined by TEGpm was highly associated with HTPR prevalence in post-PCI patients with clopidogrel administration, and played an independently predictive role in the occurrence of adverse events [25]. In addition, higher MAthrombin, shorter *R* time and higher MAadp were further demonstrated to be strongly associated with HTPR and adverse thrombotic event occurrence [26–28]. However, with respect to the aforementioned equation for TEGar calculation, a higher MAfibrin would lead to underestimation of TEGar and lower MAfibrin would cause overestimation of TEGar if the levels of MAthrombin and MAadp were unchanged. Consequently, basing on the responsiveness to antiplatelet treatment assessed by TEGpm assay, escalating antiplatelet therapy or deescalating intervention might be administrated if personalized antiplatelet regimens were employed. Although platelet and fibrin contribute to the clot strength synergistically during the dynamic thrombotic process, the global aggregation measuring approach is usually less specific to the drug action [29]. Therefore, the adjustment of antiplatelet administration should base more on the specific platelet responsiveness to drugs than total clot strength due to platelet-fibrin crosslink, such as MAadp or TEGaradp. Nonspecific antiplatelet adjustment might possibly contribute to the failure of the tailored antiplatelet strategy. Thereby, indicators specifically reflecting efficacy of antiplatelet drugs targeting to platelet are necessary to be explored and validated in clinical studies.

In the present study, with the aim to search for specific variables of TEGpm to identify HTPR and reflect antiplatelet effect, we sought to isolate the contribution of platelet to total clot strength induced by ADP (i.e.MAadp) by mathematically subtracting MAfibrin from MAadp and generated a novel derived variable, net MAadp (nMAadp). We hypothesized that nMAadp would be a potential risk indicator for HTPR with less interference, more specific to antiplatelet effect and more predictive power, and also could be used as a better reference for adjusting antiplatelet medication.

## Materials and methods

### Study population

Patients underwent PCI in China Medical University First Hospital during May 1, 2019 and November 30, 2019 were enrolled in the present study. The included patients were all administrated aspirin 75 mg per day and ticagrelor 180 mg for loading dose and 90 mg for maintaining dose. All included patients must have the tests of platelet aggregation rate induced by ADP measured by LTA (LTAaradp) and TEGpm being ordered nearly simultaneously. The qualified test records were retrospectively collected by professional member of our study team through searching and reviewing laboratory information system (LIS) and hospital information system (HIS). All the subjects included were older than 18 years and diagnosed as unstable angina or acute myocardial infarction (AMI) with the need for PCI performances. The major exclusion criteria were platelet count <100 × 10^3^/mm^3^ or >500 × 10^3^/mm^3^, and hemoglobin level <100 g/l. The study was approved by the Institutional Ethical Review Board of China Medical University First Hospital. Written informed consent was obtained before the study.

### Measurement of Ltaaradp

Venous blood samples were drawn into 3.2% sodium citrate contained tubes (Becton-Dickinson, San Jose, CA, U.S.A.). Then, the tubes were centrifuged at 120 ***g*** for 5 min to obtain platelet rich plasma (PRP) and the remained blood samples were further centrifuged at 1200 ***g*** for 10 min to recover platelet poor plasma (PPP). The PRP and PPP were stored at room temperature to be used within 2 h. Platelet aggregation was assessed at 37°C by using AggRam aggregometer (Helena Laboratories, Corp., Beaumont, TX, U.S.A.). After stimulated with 5 µmol/l ADP, LTAaradp was assessed. LTAaradp was expressed as the maximum percent change of light transmittance compared with baseline light transmittance density determined by PPP.

### Measurements of variables of TEGpm

Four TEGs (TEG5000, Haemonetics, Braintree, MA, U.S.A.) with automated analytical software were used for determining variables of *R* time, MAthrombin, MAadp and MAfibrin. Venous blood samples were drawn into two tubes with heparin and sodium citrate anticoagulant respectively. For *R* time and MAthrombin, 340 µl of citrated blood samples were used for running a kaolin-activated program with addition of 20 µl of 0.2 mol/l calcium chloride. For MAadp and MAfibrin detection, ActivatiorF reagent (constitute of reptilase and FXIIIa) and ADP were prepared by reconstitution with distilled water before testing according to manufacturer instructions. For generating MAfibrin, 360 µl of heparinized whole blood sample with 10 µl of ActivatorF only was loaded into testing cup to produce a clot due only to fibrin cross linking. MAadp was determined by loading 360 µl of heparinized whole blood sample with 10 µl of ADP and 10 µl of ActivatorF into test cup to generate the clot due to crosslink of fibrin and platelet, detailed information was described previously elsewhere [25,27]. To generate nMAadp, a derived parameter defined as the clot strength being contributed only by platelet was calculated by mathematically subtracting MAfibrin from MAadp (Figure 1). Aggregation rate induced by ADP (TEGaradp) was calculated according to the equation followed:

$$\text{TEGaradp }=\frac{\left(\text{MAadp}\;\text{-}\;\text{MAfibrin}\right)}{\left(\text{MAthrombin}\;\text{-}\;\text{MAfibrin}\right)}\times 100$$

as illustrated in Figure 1.

### Definition for HTPR and nHTPR

According to the suggestion of previous study [5], patients with more than 46% LTAaradp induced by 5 µmol/l ADP was defined as HTPR and less than or same as 46% LTAaradp was classified into nHTPR group.

### Statistical analysis

For all the analyses, MedCalc Statistical Software version 19.1 (MedCalc Software bv, Ostend, Belgium) and GraphPad Prism version 8.0.0 (GraphPad Software, San Diego, California U.S.A.) were used for appropriate tests. Continuous variable was expressed as mean ± standard deviation (SD) and compared by using the Student’s *t*-test if normal distribution was observed, otherwise rank sum test was used. For correlation tests, spearman correlation tests were used. Coefficients of each pair of comparison were tested for statistical significance by performing *r*-to-*z* transformation and *z*-test analyses. Receiver operating characteristic (ROC) curve analyses were performed to determine the cutoff values for identifying HTPR of TEGaradp, MAadp and the derived nMAadp according to the reference HTPR defined by >46% LTAaradp as recommended by the consensus paper [5]; areas under the ROC curve (AUC) were used to compare diagnostic abilities of the tested variables. To determine the predictive power of TEGpm variables measured in this study for HTPR, multiple logistic regressions were performed; Odds ratios and combined ROC curve analyses of independent variables were also analyzed. For all tests, *P*<0.05 with two tails was considered as statistical significance.

## Results

### Patient characteristics

A total of 110 patients underwent PCI with DAPT were included in the present study. All the included patients were ordered examinations of platelet reactivity with LTA and TEGpm induced by ADP nearly simultaneously. Basic characteristics of patients enrolled and relevant analyses are presented in Table 1. Briefly, based on the suggested 46% LTAaradp as cutoff value for HTPR [5], 23 (20.9%) of the 110 patients were estimated as HTPR. Age of the total enrolled subjects was 60.5 (12.3) years with 63.7 (11.3) years for HTPR vs. 59.6 (12.5) years for nHTPR (*P*=0.1656). Thirty-one enrolled subjects were female with thirteen of whom being estimated as HTPR (41.9% of female vs. 12.7% of male, *P*<0.0007). Considering of the higher prevalence of HTPR in female might be led by age influence, further analyses were performed through stratifying age variable by gender. Consequently, female patients exhibited more advanced age with 67.8 (9.5) years compared with male patient with 57.6 (12.1) years (*P*<0.001); in addition, the female patients with HTPR are also significantly older than the male with HTPR (69.7 (9.5) vs. 55.8 (8.4), *P*=0.0015). As for clinical presentation, HTPR showed more prevalent in unstable angina patient than in AMI (38.7% vs. 13.9%, *P*=0.0042). While smoking, diabetes, hyperlipidemia and hs-CRP did not show significant difference between HTPR and nHTPR groups.

**Table 1:** Characteristics of patients enrolled after percutaneous coronary intervention

	Total (*N*=110)	HTPR (23,20.9%)	nHTPR (87,79.1%)	*P* value
**Age**				
Age, mean years (SD)	60.5 (12.3)	63.7 (11.3)	59.6 (12.5)	0.1656
Male	57.6 (12.1)^†^	55.8 (8.4)	57.8 (12.6)	<0.0010
Female	67.8 (9.5)	69.7 (9.5)^‡^	66.6 (9.6)	0.0015
**Gender**				
Male, *n* (%)	79.0 (71.8)	10.0 (12.7)	69.0 (87.3)	
Female, *n* (%)	31.0 (28.2)	13.0 (41.9)^*^	18.0 (58.1)	<0.0007
**Risk factors**				
Smoking, *n* (%)	67.0 (60.1)	12.0 (17.9)	55.0 (82.1)	0.3366
Diabetes	29.0 (26.4)	6.0 (20.7)	23.0 (79.3)	0.9731
Hypertension, *n* (%)	54.0 (49.1)	9.0 (16.7)	45.0 (83.3)	0.2848
Hyperlipidemia, *n* (%)	67.0 (60.9)	12.0 (17.9)	55.0 (82.1)	0.3366
**Diagnosis**				
Unstable angina, *n* (%)	31.0 (28.2)	12.0 (38.7)	19.0 (61.3)	
AMI, *n* (%)	79.0 (71.8)	11.0 (13.9)	68.0 (86.1)	0.0042
**Laboratory data**				
WBC, ×10^3^/mm^3^ mean (SD)	7.8 (3.6)	6.8 (2.1)	8.0 (3.9)	0.1520
Platelet, ×10^3^/mm^3^ mean (SD)	210.7 (52.9)	213.0 (46.5)	210.1 (54.8)	0.8148
PWD, mean (SD)	13.9 (12.1)	12.8 (1.8)	13.6 (1.3)	0.6389
MPV, mean (SD)	10.9 (2.0)	10.8 (0.7)	10.9 (2.2)	0.8024
PM, mean (SD)	2276.6 (627.7)	2306.4 (547.4)	2268.7 (649.9)	0.7989
LPR, mean (SD)	30.7 (7.4)	31.4 (6.1)	30.5 (7.8)	0.6145
RBC, ×10^6^/mm^3^ mean (SD)	4.4 (0.6)	4.2 (0.6)	4.5 (0.6)	0.0588
HB mean, g/l (SD)	133.4 (20.2)	128.7 (17.4)	134.7 (20.8)	0.2028
Hs-CRP, mg/l, median (percentile^25–75^)	3.2 (1.5–8.6)	2.4 (1.5–5.5)	3.6 (1.7–9.0)	0.1151
ALT, U/l mean (SD)	38.0 (25.5)	35.3 (23.1)	38.7 (26.2)	0.5723
Cr, µmol/l mean (SD)	67.5 (14.7)	66.3 (14.1)	67.8 (14.9)	0.6772

HTPR was defined by LTAaradp (ADP induced aggregation rate by light transmittance aggregometry) >46%;

* indicates significant difference between gender with regarding to HTPR;

‡ indicates significant difference between ages of different gender with regarding to HTPR;

† indicates significant difference of age between gender;

Abbreviations: LPR, large platelet ratio; MPV, mean platelet volume; PM, platelet count × MPV; PWD, platelet width of distribution.

### Correlations between variables of TEGpm and LTAaradp

**Figure 2:**
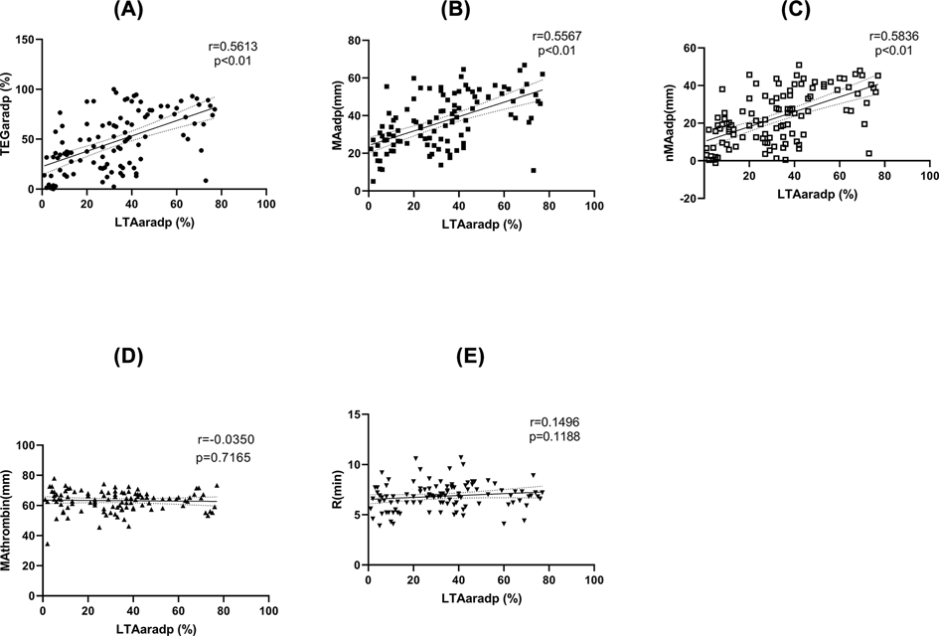
Correlations between TEGpm variables and LTAaradp TEGpm, thromboelastography with platelet mapping; TEGaradp, percentage of platelet aggregation rate induced by adenosine diphosphate (ADP) detected by TEGpm; MAthrombin, maximum amplitude (MA) induced by thrombin; MAadp, MA induced by ADP; MAfibrin, MA due to fibrin crosslink; *R*, coagulating factors reaction time indicating thrombin generation; nMAadp, net MAadp; LTA, light transmittance aggregometry; LTAaradp, percentage of platelet aggregation rate induced by ADP detected by LTA.

Table 2 and Figure 2 show the correlations between variables of TEGpm and LTAaradp. MAadp, TEGaradp and nMAadp showed gradually increased significant correlations with LTAaradp, but were all moderate (*r* = 0.5567, 0.5613 and 0.5836, respectively); by further analyzing the three coefficients with *r*-to-*z* transformations and performing *z*-tests, no significant differences were found between nMAadp and TEGaradp or MAadp (*z*=0.2427, *P*=0.8083 and *z*=0.2916, *P*=0.7706, respectively). In addition, no significant correlations were found between MAthrombin and R with LTAaradp.

**Table 2:** Correlation coefficients of between TEGmp varibles and LTAaradp

Variable	LTAaradp	TEGaradp	MAadp	nMAadp	MAtrombin
TEGaradp	0.5613				
MAadp	0.5567	0.9344			
nMAadp	0.5836	0.9713	0.9637		
MAtrombin	−0.03501	−0.1617	0.1313	−0.04935	
*R*	0.1496	0.06208	−0.01125	0.003585	−0.2459

LTAaradp, ADP induced aggregation rate by light transmittance aggregometry; TEGaradp, ADP induced aggregation rate by TEGmp; MAadp, ADP induced MA (maximum aplitude) by TEGmp; MAthrombin, thrombin induced MA by TEGmp; nMAadp, net ADP induced MA by TEGmp; Rck, kaolin activated *R* time by TEGmp.

### Comparisons of TEGpm variables between HTPR and nHTPR

**Figure 3:**
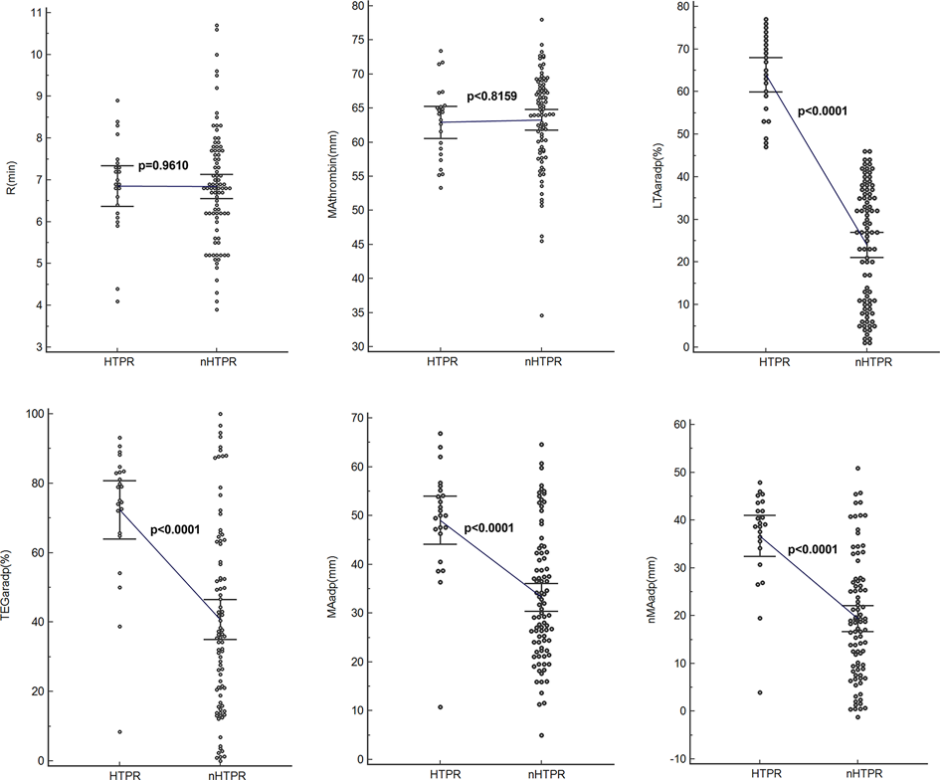
Comparisons of variables between HTPR and nHTPR determined by LTA and TEGpm TEGpm, thromboelastography with platelet mapping; TEGaradp, percentage of platelet aggregation rate induced by adenosine diphosphate (ADP) detected by TEGpm; MAthrombin, maximum amplitude (MA) induced by thrombin; MAadp, MA induced by ADP; MAfibrin, MA due to fibrin crosslink; *R*: time of coagulating factor reaction indicating thrombin generation; nMAadp, net MAadp; LTA, light transmittance aggregometry; LTAaradp, percentage of platelet aggregation rate induced by ADP detected by LTA.

Comparisons of TEGpm variables and LTAaradp between HTPR and nHTPR are depicted in Table 3 and shown in Figure 3. In detail, significant higher LTAaradp, TEGaradp, MAadp and nMAadp were demonstrated in patients with HTPR than in patient without HTPR, with 63.95%, 72.36%, 49.02 mm and 36.69 mm vs. 24.01%, 40.72%, 33.24 mm and 19.36 mm, respectively (*P*<0.0001 for all). Nevertheless, MAthrombin and R were not significantly different between patients with and without HTPR.

**Table 3:** Comparisons of TEGmp variables between HTPR and nHTPR

Variable, (unit)	HTPR (*n*=23)		nHTPR (*n*=87)		P^*^
	Mean	SD	Mean	SD	
LTAaradp, (%)	63.95	9.38	24.01	13.87	<0.0001
TEGaradp, (%)	72.36	19.30	40.72	26.98	<0.0001
MAadp, (mm)	49.02	11.39	33.24	13.24	<0.0001
MAthrombin, (mm)	62.92	5.49	63.29	7.10	0.8159
nMAadp, (mm)	36.69	9.95	19.36	12.81	<0.0001
*R* (min)	6.85	1.13	6.84	1.36	0.9610

LTAaradp, ADP induced aggregation rate by light transmittance aggregometry; TEGaradp, ADP induced aggregation rate by TEGmp; MAadp, ADP induced MA (maximum aplitude) by TEGmp; MAthrombin, thrombin induced MA by TEGmp; nMAadp, net ADP induced MA by TEGmp; *R*, kaolin activated R time by TEGmp. P*: *t*-test with *P* value less than 0.05.

### Comparisons of areas under the receiver operating characteristic curve (AUC) of TEGpm in identification of HTPR

**Figure 4:**
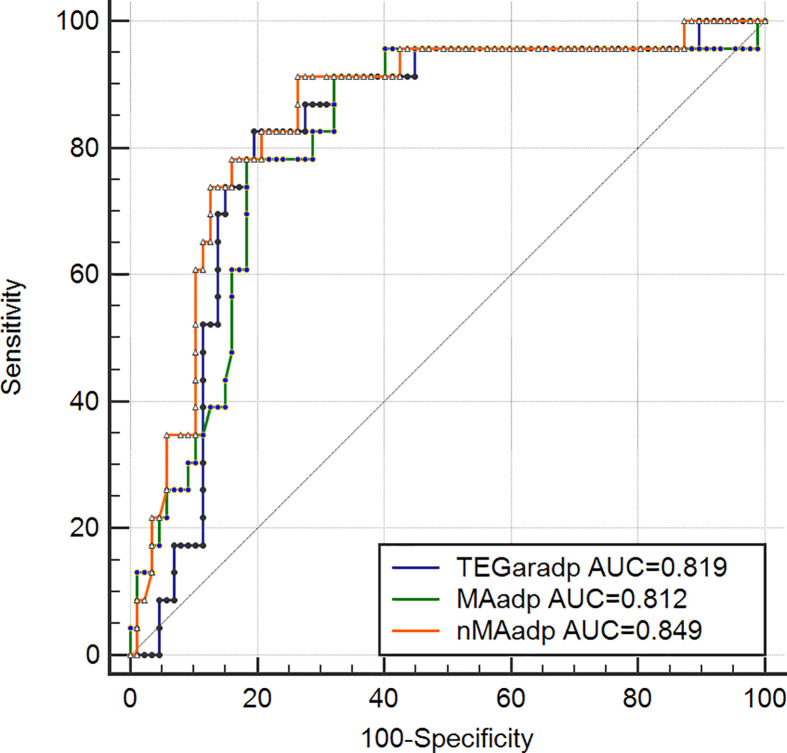
Receiver operating characteristic (ROC) curves of TEGpm variables for HTPR TEGpm, thromboelastography with platelet mapping; TEGaradp, percentage of platelet aggregation rate induced by adenosine diphosphate (ADP) detected by TEGpm; MAadp, maximum amplitude (MA) induced by ADP; nMAadp, net MAadp; LTAaradp, percentage of platelet aggregation rate induced by ADP; HTPR, high on treatment platelet reactivity; ROC analyses based on HTPR defined by LTAaradp >46%. LTAaradp: percentage of platelet aggregation rate induced by ADP detected by LTA.

Based on LTAaradp >46% defined cutoff value for HTPR, variables of TEGpm with significant correlation to LTAaradp were selected and tested by using receiver operating characteristic curve (ROC) analyses and correspondent cutoff values for identification of HTPR were calculated, as shown in Table 4 and Figure 4. The AUCs of nMAadp, MAadp and TEGaradp were 0.849, 0.812 and 0.819 respectively. Difference between AUC of nMAadp and AUCs of MAadp and TEGaradp were statistically significant (*P* = 0.0098 and 0.0429). Thus, nMAadp was the most powerful indicator of HTPR. Cutoff values of nMAadp, TEGaradp and MAadp were calculated as 26.3 mm (sensitivity 91.30%, specificity 73.56%, positive likelihood ratio [LR+] 3.45 and negative likelihood ratio[LR-] 0.12), 64.6% (sensitivity 82.61%, specificity 80.46%, LR+ 4.23 and LR- 0.22) and 45.4 mm (sensitivity 78.26%, specificity 81.61%, LR+ 4.26 and LR- 0.27), respectively.

**Table 4:** Comparisons of AUC of TEGmp variables based on HTPR defined by LTAaradp

Variable	AUC	95% CI	Cutoff	Sensitivity	Specificity	LR+	LR-	*P* value
TEGaradp (%)	0.819	0.734 to 0.886	64.6	82.61	80.46	4.23	0.22	0.0429
MAadp (mm)	0.812	0.726 to 0.880	45.4	78.26	81.61	4.26	0.27	0.0098
nMAadp (mm)	0.849	0.768 to 0.910	26.3	91.30	73.56	3.45	0.12	

LTAaradp, ADP induced aggregation rate by light transmittance aggregometry; TEGaradp, ADP induced aggregation rate by TEGmp; MAadp, ADP induced MA (maximum aplitude) by TEGmp; nMAadp, net ADP induced MA by TEGmp; LR+, positive likelihood ratio; LR-, negative likelihood ratio; *P* value indicates AUC of TEGaradp and MAadp compared to nMAadp; AUC, area under the receiver operating curve.

### Multivariable logistic regression model

**Figure 5:**
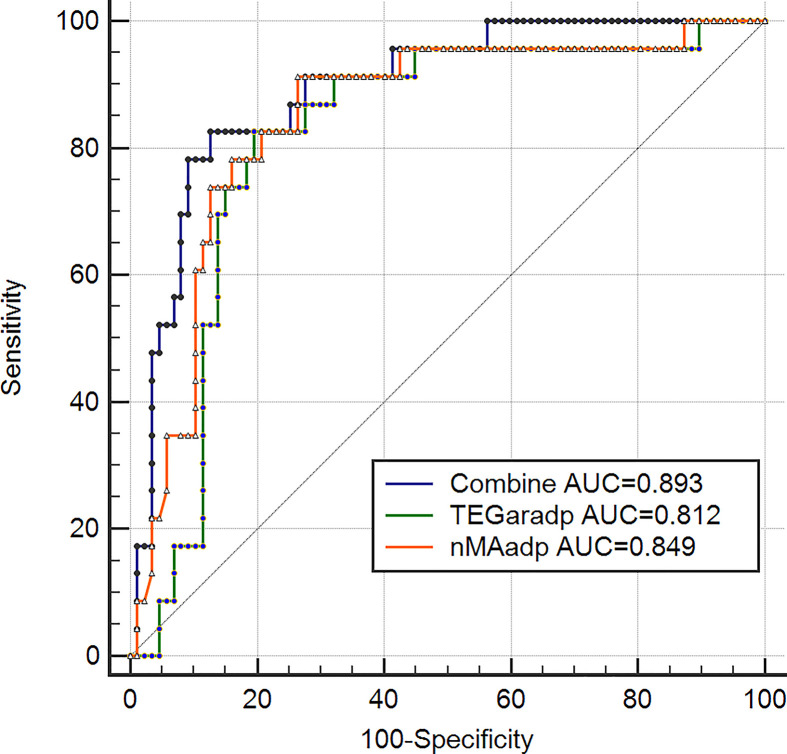
Combined receiver operating characteristic (ROC) curves of TEGpm variables for HTPR TEGpm, thromboelastography with platelet mapping; TEGaradp, percentage of platelet aggregation rate induced by adenosine diphosphate (ADP) detected by TEGpm; nMAadp, net maximum amplitude induced by ADP; combine, combination of nMAadp and TEGaradp; LTAaradp, percentage of platelet aggregation rate induced by ADP; HTPR, high on treatment platelet reactivity; ROC analyses based on HTPR defined by LTAaradp >46%. LTAaradp, percentage of platelet aggregation rate induced by ADP detected by LTA.

All the measured TEGpm variables were put into multiple logistic regression models. After adjusting for confounding factors, nMAadp and TEGaradp were demonstrated to be the independent indicators for predicting HTPR, the odds ratios (OR) of nMAadp and TEGaradp were 1.9554 and 0.7825, respectively (Table 5). In addition, combined capacity of nMAadp and TEGaradp to predict HTPR was determined with an AUC of 0.893, as shown in Figure 5.

**Table 5:** Logistic regression of TEGmp variables based on HTPR

Variable	Coefficient	Odds ratio	Wald	*P*
TEGaradp	−0.24527	0.7825	4.7785	0.0288*
MAadp	−0.047911	0.9532	0.08264	0.7738
MAtrombin	−0.13672	0.8722	1.0818	0.2983
nMAadp	0.67061	1.9554	11.0952	0.0009*
*R*	0.37568	1.4560	1.9139	0.1665

HTPR was defined by LTAaradp (ADP induced aggregation rate by light transmittance aggregometry) >46%; TEGaradp: ADP induced aggregation rate by TEGmp; MAadp: ADP induced MA (maximum aplitude) by TEGmp; MAthrombin: thrombin induced MA by TEGmp; nMAadp: net ADP induced MA by TEGmp; *R*: kaolin activated R time by TEGmp.

* indicates statistical significant.

## Discussion

In the present study, a derived TEGpm variable, nMAadp was demonstrated to be as a novel independent risk indicator with more power to identify HTPR in post-PCI patients and the cutoff value of nMAadp for HTPR was calculated as more than 26.3 mm. Moreover, TEGaradp was also demonstrated to be more powerful than MAadp in discriminating patients with or without HTPR. Combination of nMAadp and TEGaradp further increased ability to identify HTPR. While MAthrombin and *R* time of TEGpm assay were not significantly different between patients with and without HTPR.

Hemostatic plug formation is a complex and dynamic process with critical contribution of platelet activity [1,3,30]. HTPR is strongly associated with thrombotic event occurrence in patients with ACS and patients undergoing PCI [5,31]. Over the past decades, antiplatelet treatment was widely adopted aiming to reduce the TPR and decrease the risk of thrombotic event occurrence, although personalized antiplatelet strategy based on PFTs did not see significant benefits in multiple studies [8–10,29,32]. So far, PFTs are currently not recommended in routine clinical practice due at least part to unsuccessful improvement of tailored antiplatelet therapy based on PFT guidance [4,29]. Numerous factors might contribute to the failure of PFT guided tailored antiplatelet therapy. Currently, there is no uniform standard for PFT in assessing platelet reactivity; various available PFTs are now being applied in studies and clinical uses, which might cause considerable variations between assays; in addition, indicators of PFTs for reflecting responsiveness to antiplatelet medication or TPR may not be specific enough for guiding successful personalized therapy. Thus, seeking for universally recognized PFTs and specific indicators to identify HTPR and stratify risks of adverse event occurrence for improving tailor antiplatelet therapy are of great importance.

An ideal PFT should be reliable, accurate, easy to operate and available with flexible time at bedside [13]. So far, available PFTs can be mainly classified into laboratory based test and point-of-care test [12,13]. LTA is a traditionally laboratory based test and was developed for evaluating platelet reactivity half a century ago [14]. LTA has been historically considered as a gold standard test to evaluate platelet reactivity in numerous studies [26–28,33]. However, LTA method is challenged for lacking of standardization, time-consuming and complicated sample preparation, which limits its clinical routine [12]. As recommended by recent updated consensus suggestion regarding the clinical practicality, POCTs for platelet reactivity assessment should be more preferred [31]. Therefore, of the currently available POCTs for platelet reactivity evaluation, the TEGpm meets the practical criteria and gradually raises the interests for exploration [26–28,34].

Comparisons of various currently used PFTs have been extensively studied. Variations and poor concordances of different PFTs were demonstrated [17,35]. Before adopting a new PFT to be applied in clinics, correlation analysis between the new one and the gold standard method is generally performed. In our study, TEGpm variables, including TEGaradp, MAadp and the newly derived nMAadp, were demonstrated to be moderate but significant correlated with LTAaradp. nMAadp was the strongest correlated variable (*r*=0.5836) to LTAaradp compared with TEGaradp (*r*=0.5613) and MAadp (*r*=0.5567), but statistical significance of correlation coefficients among them were not obtained by performing *z*-test analyses with *r-*to-*z* transformation. This was not well in line with the result of a study, which reported the strongest correlation between LTAaradp and TEGaradp with a correlation coefficient of 0.821 [25]; the other studies employed TEGpm assay and LTA method showed moderate correlations between TEGaradp and LTAaradp, with 0.675 to 0.733 of correlation coefficient [33,36]. The differences between the results of our study with them may be due to different antiplatelet drug used, various subjects enrolled and various devices employed.

Over past decade, various TEG variables have been explored and demonstrated be capable to indentify HTPR and stratify risks of adverse event occurrence in post-PCI patients. MAthrombin and R time were reported by Gubel et al. to be as predictive indicators for HTPR and could potentially predict adverse post-PCI events occurrence [28]. Furthermore, a delayed *R* time after treatment with clopidogrel compared with pre-treatment baseline was demonstrated to be directly correlated with the dose-related effect of antiplatelet treatment [26]. More recently, a study of 225 enrolled patients after elective PCI treated with aspirin and clopidogrel demonstrated that MAadp was an independent risk predictor not only for ischemic event occurrence but also for bleeding event, the cutoff value of MAadp for ischemic event occurrence was estimated more than 47 mm [27], which was accordingly suggested as HTPR threshold and the upper limit of therapeutic window for antiplatelet strategy [5]. In our study, we examined the ability of MAthrombin, R time, MAadp, TEGaradp and nMAadp to identify HTPR in post-PCI patients. It was surprisingly found that MAthrombin and *R* time were not significantly different between patients with and without HTPR. Reasonable explanations of the contradiction to the results reported by Gurbel et al. might be the two variables were not specific enough to identify HTPR, or the relatively less number of subjects included in our study did not allow the statistical significance to be concluded [26,28]; in addition, we used HTPR defined by LTAaradp as reference outcome, but not clinical endpoints of adverse event occurrence. By using ROC analyses to determine the ability of TEGpm variables for discriminating HTPR in our study, TEGaradp, MAadp and nMAadp all yielded satisfactory and significant AUC for identifying HTPR. Of these three TEGpm variables examined, nMAadp yielded an AUC of 0.849 followed by TEGaradp and MAadp with 0.819 and 0.812, respectively. nMAadp was demonstrated to be the strongest indicator for HTPR compared with TEGaradp and MAadp (*P* = 0.0429 and 0.0098, respectively). Furthermore, cutoff values of nMAadp, MAadp and TEGaradp for HTPR were calculated as 26.3 mm, 45.4 mm and 64.6% respectively. In the study of Gulbel et al., 47 mm of MAadp was derived as cutoff value for prediction of thrombotic event occurrence in patients undergone PCI [27], which was identical to the result of the other most recent report that recruited Chinese patients with PCI procedures [37]. In one other study also with LTAaradp as reference, the cutoff value of MAadp for predicting ischemic event occurrence was reported as 47.5 mm [36]. However, when a whole blood sample based assay for platelet function evaluation, VerifyNow was used as reference for determining HTPR, 45.2 mm was derived as MAadp cutoff value for HTPR, which was very similar to the result of 45.4 mm of MAadp reported in our study. The disagreement of our result to the above-mentioned reports, however, further confirmed the highly existing variation and low concordance of inter-assay analyses and inter-laboratory assays, but also prompted the urgent need for standardization of PFTs, application of universally recognized methods as well as establishment of uniform reference values of risk indicators. For selecting independent indicators for HTPR, multivariable logistic model was used in our study through entering the potential variables measured by TEGpm assay, including nMAadp, TEGaradp and MAadp. As expected, nMAadp was demonstrated to be a significantly independent indicator for HTPR with an OR of 1.9554. TEGaradp was also contributed independently and significantly to HTPR with an OR of 0.7825. However, MAadp was removed from the model for inability to HTPR discrimination. Reasonable explanations might be the relatively strong colinearity of MAadp and TEGaradp, but TEGaradp contributes much more to the HTPR identification than MAadp; in addition, the level of MAadp does not reflect the clot strength due only to platelet contribution but also affected by the fibrinogen level, which might possibly reduce the indicative power of evaluating the responsiveness to antiplatelet treatment in terms of specific targets of platelet inhibitors. Therefore, nMAadp, which reflects the extent of clot strength mathematically attributable to platelet alone, gained stronger ability to evaluate platelet reactivity than MAadp. Furthermore, it is worth to note that the calculating equation of TEGar, such as TEGaradp, has its advantage for not only consideration of the reduction of platelet aggregation ability induced by ADP but also taking baseline MAthrombin level into account. Higher MAthrombin produces higher TEGaradp under the same MAfibrin and MAadp levels, which asks for more enhanced antiplatelet strategy even under the same reductive extent of MAadp compared with MAthrombin. Thus, taking specificity of antiplatelet effect and baseline platelet reactivity into account together, it might be reasonable to consider that more predictive information on antiplatelet efficacy would be yielded by using the combination of TEGaradp and nMAadp together for HTPR identification. As expected, by using a combined ROC analysis, the combination of TEGaradp with nMAadp yielded an AUC of 0.893 to predict HTPR and might promisingly be considered as a reliable reference for identification of high risk patients with DAPT after PCI.

In conclusion, we reported nMAadp as a novel independent indicator for predicting HTPR, and demonstrated TEGaradp was more indicative than MAadp in discriminating HTPR as well as further confirmed the highly existing variations of inter-assay and inter-laboratory assay for platelet reactivity examinations. However, several limitations of our study are deserved to be mentioned. First, HTPR of patients undergone PCI in our study was defined by plasma sample based LTA method, but not by other available POCTs using whole blood sample, such as VerifyNow assay, which may be more alike to the detecting milieu of TEGpm. Second, a relatively less number of subjects were enrolled in our study, which might prevent some statistical significance from being obtained. But, a serial of analyses with a hundred and ten subjects included in our study indeed provided useful information for appraising the ability of TEGpm variables to identify HTPR in post-PCI patients. Third, the subjects enrolled in the present study were received DAPT with aspirin and ticagrelor, but not with other P2Y_12_ inhibitors such as clopidogrel, prasugrel and cangrelor, which needs further evaluation in terms of prediction and identification of HTPR by TEGpm variables. Finally, the present study was a retrospective study, which might possibly cause analytical bias. Future prospective clinical trials with larger number of participants and objective clinical endpoints of major adverse event occurrence are needed to provide robust evidence for validating the usefulness of nMAadp in evaluating platelet reactivity and guiding tailored antiplatelet treatment.

## Abbreviations

HTPR: high-on treatment platelet reactivity
LTA: light transmittance aggregometry
MA: maximum amplitude
OR: odds ratios
PCI: percutaneous coronary interventions
PFA: platelet function analyzer
PFT: platelet function test
POCT: point-of-care test
ROC: receiver operating characteristic
TEG: thromboelastography
TEGpm: thromboelastograph with platelet mapping
VASP: vasodilator-stimulated phosphoprotein
